# Public Views About Opioid Overdose and People With Opioid Use Disorder

**DOI:** 10.1001/jamanetworkopen.2025.54314

**Published:** 2026-01-16

**Authors:** Emma E. McGinty, Sarah Gutkind, Jeff Niederdeppe, Erika Franklin Fowler, Colleen L. Barry

**Affiliations:** 1 Division of Health Policy & Economics, Cornell Health Policy Center, Weill Cornell Medical College, New York, New York; 2 Division of Health Policy & Economics, Weill Cornell Medical College, New York, New York; 3 Department of Communication, Cornell Health Policy Center, Cornell University, Ithaca, New York; 4 Department of Government, Wesleyan University, Middletown, Connecticut; 5 Jeb E. Brooks School of Public Policy, Cornell Health Policy Center, Cornell University, Ithaca, New York

## Abstract

**Importance:**

Opioid overdoses in the US began to decline in 2024, concurrent with the transition to a new federal administration that will shape how the government prioritizes and responds to opioid overdose in coming years. Understanding US residents’ current views on opioid overdose and addiction can inform future efforts to address these issues.

**Objective:**

To examine how US adults view opioid overdose and people with opioid addiction in 2025, and how views vary by conservative, moderate, or liberal ideology.

**Design, Setting, and Participants:**

This web-based survey of a national sample of adults who identified their race and ethnicity as non-Hispanic or Hispanic Black or non-Hispanic White was fielded April 7 to 28, 2025.

**Main Outcomes and Measures:**

Respondents rated their perceptions of the seriousness of opioid overdose deaths in the US; the degree to which they viewed people who use opioids, their family members, pharmaceutical companies, governments (federal, state, and local), or nonprofits as responsible for reducing overdose deaths; and social distance attitudes (willingness to have a person with opioid addiction as a neighbor or marry into their family).

**Results:**

Among the 1552 survey respondents (939 female [60.5%]; 523 aged 30-44 years [33.7%]; 756 non-Hispanic or Hispanic Black [48.7%]; 796 non-Hispanic White [51.3%]), 448 (28.9%) politically identified as conservatives, 615 (39.6%) as moderates, and 489 (31.5%) as liberals. Overall, 88.2% (95% CI, 86.0%-90.1%) of adults viewed opioid overdose deaths as serious, as did 83.4% (95% CI, 78.8%-87.1%) of conservatives, 88.7% (95% CI, 85.2%-91.5%) of moderates, and 93.4% (95% CI, 90.4%-95.6%) of liberals. Respondents viewed people who use opioids (81.0%; 95% CI, 78.4%-83.4%) and pharmaceutical companies (72.7%; 95% CI, 69.7%-75.4%) as most responsible for reducing overdose deaths. More liberals (83.4%; 95% CI, 78.6%-87.3%) identified pharmaceutical companies as responsible than people who use opioids (69.6%; 95% CI, 64.0%-74.3%). Regarding social distance, 38.3% (95% CI, 35.3%-41.5%) of respondents were unwilling to have a person with opioid addiction as a neighbor and 58.4% (95% CI, 38.5%-61.5%) were unwilling to have a person with opioid addiction marry into their family. Desire for social distance was greater among conservatives (neighbor: 52.0%; 95% CI, 46.5%-57.4%; marry: 71.0%; 95% CI, 65.9%-75.7%) vs moderates (neighbor: 34.0%; 95% CI, 29.2%-39.2%; marry: 54.4%; 95% CI, 49.2%-59.5%) or liberals (neighbor: 27.0%; 95% CI, 22.3%-32.4%; marry: 48.0%; 95% CI, 42.3%-53.7%).

**Conclusions and Relevance:**

In this 2025 survey study, US adults viewed opioid overdose as a serious problem. Different views on the degree to which individuals who use opioids, pharmaceutical companies, and governments are responsible for reducing overdose suggest that preferences for future actions to address overdose may vary among conservatives, moderates, and liberals.

## Key Points

**Question:**

How do US adults view the severity of the opioid overdose problem, responsibility for solving it, and people experiencing opioid addiction, and do views differ by political ideology?

**Findings:**

In this survey study of 1552 US adults, more than 70% of survey respondents viewed opioid overdose as serious and identified people who use opioids and pharmaceutical companies as most responsible for solutions; by ideology, 65% or more of conservatives, moderates, and liberals shared these views. Overall, 38% and 58% were unwilling to have a person with opioid addiction as a neighbor or marry into their family, respectively.

**Meaning:**

These findings suggest that the US public continues to view opioid overdose as a serious and stigmatized condition.

## Teaser

This survey study examines how US adults view opioid overdose and people with opioid addiction and how views vary by conservative, moderate, or liberal ideology.

## Introduction

Over the past decade, US adults and the federal government have viewed rising rates of opioid overdose as a public health crisis.^1^ However, for the first time in over 2 decades, US opioid overdose deaths meaningfully decreased from 83 140 deaths in 2023 to 54 743 deaths in 2024.^2^ This decline coincides with a new federal administration that will shape how the US government prioritizes and responds to opioid overdose in coming years.

Since the onset of the US opioid overdose crisis in the late 1990s, the US has shifted from a predominantly criminal-legal approach targeting drug distribution and use toward a more medical and public health–oriented approach encompassing prevention, treatment, and harm reduction.^3^ The new federal administration has reemphasized efforts to curb illicit drug trafficking, particularly of fentanyl, and pledged to end funding for some harm reduction initiatives.^4^ How other priorities and strategies may shift remains to be seen, with early indications of continued investment in prevention and treatment approaches.^5^

In light of these epidemiologic and political shifts, it is important to understand how US residents view the severity of the opioid overdose problem, responsibility for solving it, people experiencing opioid addiction, and how these views may differ by political ideology. Research shows that these dimensions influence preferences for solutions to opioid overdose^6,7^ and has identified differences in views across political ideologies.^8,9^ Pervasive views of drug use as a moral failing have led many US residents to blame people who use opioids for the overdose problem, contributing to negative attitudes toward this group.^10,11^ These views have not been comprehensively assessed in the 2020s. This study fills that gap with a survey conducted in April 2025.

## Methods

This survey study was deemed exempt by the Cornell University institutional review board due to the use of deidentified data in accordance with the Common Rule 45 CFR 46. This study is reported in alignment with the Checklist for Reporting of Survey Studies (CROSS) and American Association for Public Opinion Research (AAPOR) reporting guideline.

### Study Design and Participants

We conducted a web survey with a national sample of adults aged 18 years or older who identified as Hispanic Black, non-Hispanic Back, and non-Hispanic White. Race and ethnicity were self-reported using categorical options. For race, these included Asian, Black or African American, Native Hawaiian or Other Pacific Islander, White, and another race. For ethnicity, respondents were asked, “Are you of Hispanic, Latino/a/x, or Spanish origin?” Individuals who identified as non-Hispanic White or non-Hispanic or Hispanic Black were eligible for the study.

The survey was fielded from April 7 to 28, 2025, using the SSRS Opinion Panel, a probability-based web panel. Opinion panel members are recruited via simple random sampling from 2 sample frames: the US Postal Service Computerized Delivery Sequence, an address-based sampling frame including 98% to 99% of the US population, and random digit dial samples through other SSRS surveys.^12^ The SSRS Opinion Panel recruitment rate is 6% and includes approximately 50 000 individuals with ongoing recruitment. The study sample was drawn from this panel.

Data used in this study are a subset of a larger survey-embedded message testing study. In the larger study, a simple random sample of 13 521 Black or non-Hispanic White adults from the 50 000-member SSRS Opinion Panel were invited to participate and 5623 completed the survey, for a study response rate of 44%. The AAPOR composite response rate, known as AAPOR response rate 3,^13^ which incorporates the overall Opinion Panel recruitment rate, was 2%. Of the 5623 individuals who participated in the larger survey-embedded message testing study, 4071 were randomly assigned to view a message prior to answering the survey questions. A subset of these 4071 individuals were randomly assigned to a control group that answered survey questions with no message exposure; these individuals represent this study’s sample. Further information about the overall message testing study is available on the Open Science Framework preregistration page.^14^

Prior to completing the survey, respondents viewed a brief script (CROSS checklist) describing study objectives, rewards, and confidentiality protections and noting that participation was voluntary and that respondents could skip questions they did not wish to answer. After viewing this script, respondents selected an option to complete (or not complete) the survey.

### Measures

The overall survey included 58 total items related to opioids and Medicaid, with a brief preamble introducing each topic. This study reports 4 opioid-related items. The preamble text and survey items used in the present study are included in eAppendix 1 in Supplement 1. The full questionnaire is available at the Open Science Framework preregistration page.^14^

All items used Likert scale response options. We measured the proportion of respondents agreeing with the statement, “The number of people who die from opioid overdose in the US is a very serious problem,” (1 = strongly disagree; 2 = disagree; 3 = neither agree nor disagree; 4 = agree; 5 = strongly agree) and the proportion attributing responsibility for reducing opioid overdose deaths (1 = none at all; 2 = a little bit; 3 = some; 4 = a lot; 5 = a great deal) to people who use opioids; their family members; pharmaceutical companies; federal, state, or local governments; and nonprofit organizations. Two items assessed desired social distance: “How willing would you be to have a person with opioid addiction [1] as a neighbor or [2] marry into your family” (1 = definitely willing; 2 = probably willing; 3 = neither willing nor unwilling; 4 = probably unwilling; 5 = definitely unwilling). These social distance measures are standard indicators of public stigma.^15^ To aid interpretation, we created binary measures from the 5-point Likert scale responses. Specifically, we created 1/0-coded measures that combined Likert scale categories 4 and 5 (binary measure value 1) and 1, 2, and 3 (binary measure value 0) to create indicators of agreement, a lot or a great deal of responsibility, and unwillingness.

Political ideology was measured on a 7-point Likert scale, which we collapsed following standard practice,^16,17^ into liberal (extremely liberal, liberal, and slightly liberal), moderate, and conservative (extremely conservative, conservative, and slightly conservative) categories. Finally, we measured respondent sociodemographic characteristics. Including sex, age, race and ethnicity, education, household income, employment status, region, political party affiliation, and personal experience with opioid addiction or overdose, defined as having personal experience with opioid addiction, a family member or close friend with opioid addiction, or knowing someone who died of a drug overdose. All measures were self-reported with categorical options, except age which was reported in years. See eAppendix 1 in Supplement 1 for item wording.

### Statistical Analysis

All analyses incorporated survey weights to calculate estimates designed to be nationally representative of Black and non-Hispanic White US adults. We first calculated the unweighted and weighted sociodemographic characteristics of the study sample relative to overall US population benchmarks. We then assessed the unweighted proportion of respondents endorsing each opioid measure of interest and used Pearson χ^2^ tests to assess differences in unadjusted proportions by ideology. Finally, average predicted probabilities were estimated from multivariate logistic regression models estimating the differences in each opioid-related survey measure of interest by political ideology and the other sociodemographic characteristics measured. Results of statistical tests were considered significant if the 2-sided *P* value was less than .05. Survey weighting addressed nonresponse error by accounting for the probability of recruitment into the overall SSRS Opinion Panel and the probability of selection into this study. Analyses were conducted in Stata version 17.0 (StataCorp).

## Results

Among the 1552 survey respondents, 939 (60.5%) were female, 523 (33.7%) were aged 30 to 44 years, 756 (48.7%) were non-Hispanic or Hispanic Black, 796 (51.3%) were non-Hispanic White, 629 (40.5%) had a bachelor’s degree or higher, and 694 (44.7%) had household income greater than $75 000. In regard to political ideology, 448 (28.9%) identified as conservatives, 615 (39.6%) as moderates, and 489 (31.5%) as liberals (eAppendix 2 in Supplement 1). Sociodemographic characteristics of the survey sample were similar to national estimates overall, though the unweighted survey sample had a slightly greater proportion of females, fewer young adults aged 18 to 29 years and more adults in the 30 to 44 and 45 to 59 years age groups, a lower proportion of people with household income greater than $75 000, and a higher proportion of political moderates and lower proportion of political conservatives relative to national benchmarks. Weighting narrowed but did not fully address these differences (eAppendix 2 in Supplement 1).

**Figure 1.**
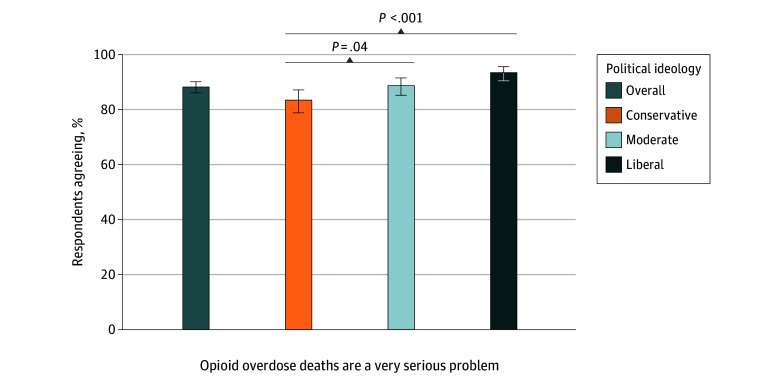
Perceived Seriousness of the Problem of Opioid Overdose Deaths Among 1552 US Adults This graph shows responses to the following item: “Please indicate your level of agreement with the following statement about opioid overdose deaths: The number of people who die from opioid overdose in the US is a very serious problem.” Response options included (1) strongly disagree, (2) disagree, (3) neither agree nor disagree, (4) agree, and (5) strongly agree. Categories 4 (agree) and 5 (strongly agree) were collapsed to indicate percentage agreement. *P *values are relative to conservatives as the reference group.

**Figure 2.**
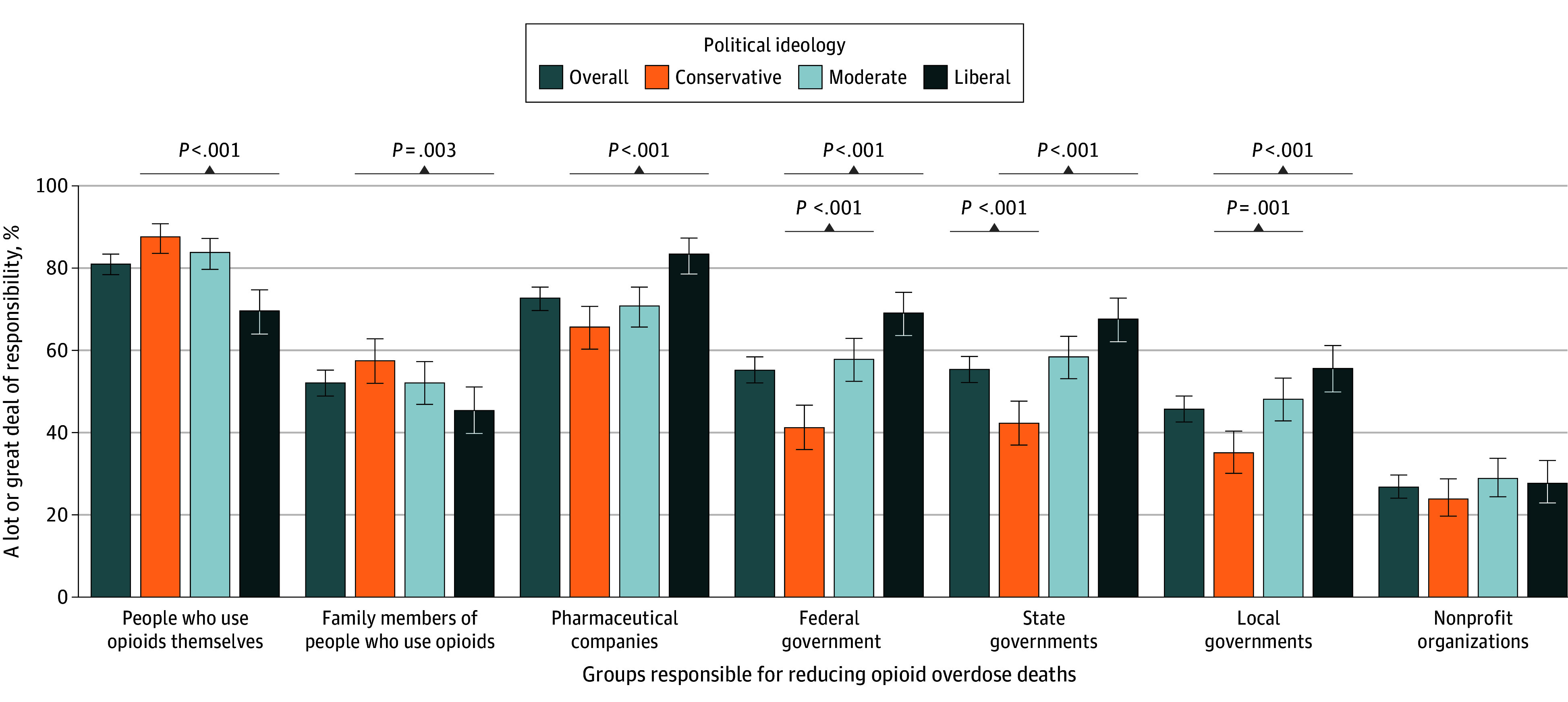
Perceived Responsibility for Reducing Opioid Overdose Deaths Among 1552 US Adults This graph shows responses to the following item: “How much responsibility do you believe each of the following groups should have for reducing opioid overdose deaths in the US: the federal government, state governments, local governments, pharmaceutical companies, charitable organizations or nonprofits, people who use opioids themselves, and family members of people who use opioids?” Response options for each group included (1) none at all, (2) a little bit, (3) some, (4) a lot, and (5) a great deal. Categories 4 (a lot) and 5 (a great deal) were collapsed to indicate percentage responsible. *P *values are relative to conservatives as the reference group.

Of all adults surveyed, 88.2% (95% CI, 86.0%-90.1%) viewed opioid overdose deaths as a very serious problem, as did more than 80% of conservatives (83.4%; 95% CI, 78.8%-87.1%), moderates (88.7%; 95% CI, 85.2%-91.5%), and liberals (93.4%; 95% CI, 90.4%-95.6%) (Figure 1). Overall, respondents viewed people who use opioids (81.0%; 95% CI, 78.4%-83.4%) and pharmaceutical companies (72.7%; 95% CI, 69.7%-75.4%) as most responsible for reducing opioid overdose deaths (Figure 2). More liberals identified pharmaceutical companies as bearing responsibility than people who use opioids, whereas more conservatives and moderates identified individuals as most responsible. Specifically, 87.6% (95% CI, 83.6%-90.8%) of conservatives, 83.8% (95% CI, 79.7%-87.2%) of moderates, and 69.6% (95% CI, 64.0%-74.7%) of liberals reported the view that people who use opioids, themselves, bear a lot or a great deal of responsibility for reducing opioid overdose deaths. Overall, 65.7% (95% CI, 60.3%-70.7%) of conservatives, 70.8% (95% CI, 65.7%-75.4%) of moderates, and 83.4% (95% CI, 78.6%-87.3%) of liberals viewed pharmaceutical companies as responsible for reducing opioid overdose.

Of all respondents, 52.1% (95% CI, 48.9%-55.2%) viewed family members of people who use opioid as responsible for reducing opioid overdose deaths, with a lower proportion of liberals (45.4%; 95% CI, 39.8%-51.1%) viewing family as responsible relative to conservatives (57.5%; 95% CI, 52.0%-62.8%); the difference between the proportion of conservatives and moderates (52.1%; 95% CI, 46.9%-57.3%) viewing family as responsible was not statistically significant. Between 45% to 55% of respondents viewed federal (55.2%; 95% CI, 52.1%-58.4%), state (55.4%; 95% CI, 52.2%-58.5%), and local (45.7%; 95% CI, 42.6%-48.9%) governments as responsible for reducing opioid overdose deaths, with higher proportions of moderates and liberals, relative to conservatives, viewing government as responsible. Overall, 26.8% (95% CI, 24.1%-29.7%) of respondents viewed nonprofit organizations as responsible, with no differences by ideology.

**Figure 3.**
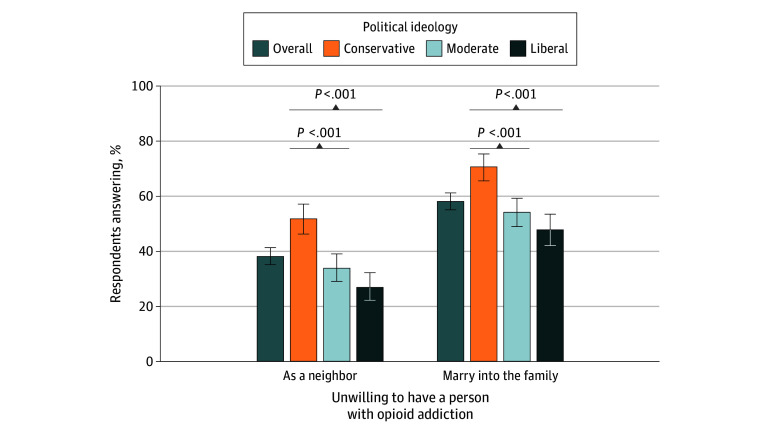
Attitudes About People With Opioid Addiction Among 1552 US Adults This graph shows responses to the following items: “How willing would you be to…have a person with opioid addiction as a neighbor [or]…have a person with opioid addiction marry into your family?” Response options included (1) definitely willing, (2) probably willing, (3) neither willing nor unwilling, (4) probably unwilling, and (5) definitely unwilling. Categories 4 (probably unwilling) and 5 (definitely unwilling) were collapsed to indicate percentage unwilling. *P *values are relative to conservatives as the reference group.

Among respondents, 38.3% (95% CI, 35.3%-41.5%) were unwilling to have a person with opioid addiction as a neighbor and 58.4% (95% CI, 55.3%-61.5%) were unwilling to have a person with opioid addiction marry into their family, with higher unwillingness on both measures among conservatives relative to moderates and liberals (Figure 3). Specifically, 52.0% (95% CI, 46.5%-57.4%) of conservatives, 34.0% (95% CI, 29.2%-39.2%) of moderates, and 27.0% (95% CI, 22.3%-32.4%) of liberals reported unwillingness to have a person with opioid addiction as a neighbor and 71.0% (95% CI, 65.9%-75.7%) of conservatives, 54.4% (95% CI, 49.2%-59.5%) of moderates, and 48.0% (95% CI, 42.3%-53.7%) of liberals reported unwillingness to have a person with opioid addiction marry into their family.

Compared with the unadjusted results, results of multivariate analyses showed similar magnitude of and differences in views among political conservatives, moderates, and liberals (Table 1 and Table 2; eAppendix 3 in Supplement 1). Adjusting for sociodemographic characteristics, an estimated 83.0% (95% CI, 78.7%-87.2%) of conservatives, 89.0% (95% CI, 86.0%-92.1%) of moderates, and 93.4% (95% CI, 90.8%-96.0%) of liberals viewed opioid overdose deaths as a very serious problem; perceived severity was greater among moderates and liberals relative to conservatives (Table 1). As in unadjusted analyses, respondents across ideological groups viewed people who use opioids and pharmaceutical companies as most responsible for reducing opioid overdose deaths, with liberals viewing pharmaceutical companies as most responsible and moderates and conservatives viewing individuals with addiction as most responsible. Specifically, adjusting for sociodemographic characteristics, an estimated 86.7% (95% CI, 83.0%-90.4%) of conservatives, 83.7% (95% CI, 79.9%-87.4%) of moderates, and 71.5% (95% CI, 66.0%-76.9%) of liberals viewed people with opioid addiction as responsible, and 66.0% (95% CI, 61.0%-71.1%) of conservatives, 71.8% (95% CI, 67.2%-76.4%) of moderates, and 82.5% (95% CI, 78.1%-86.9%) of liberals viewed pharmaceutical companies as responsible (Table 1).

**Table 1.** Adjusted Perceptions of the Seriousness of and Responsibility for Solving the Problem of Opioid Overdose Among 1552 US Adults

Characteristic	Opioid overdose deaths are a very serious problem		People who use opioids are responsible for reducing opioid overdose deaths		Pharmaceutical companies are responsible for reducing opioid overdose deaths	
	Agree, % (95% CI)	*P* value	A lot or a great deal of responsibility, % (95% CI)	*P* value	A lot or a great deal of responsibility, % (95% CI)	*P* value
Political ideology						
Conservative	83.0 (78.7-87.2)	[Reference]	86.7 (83.0-90.4)	[Reference]	66.0 (61.0-71.1)	[Reference]
Moderate	89.0 (86.0-92.1)	.03^a^	83.7 (79.9-87.4)	.26	71.8 (67.2-76.4)	.11
Liberal	93.4 (90.8-96.0)	<.001^a^	71.5 (66.0-76.9)	<.001^a^	82.5 (78.1-86.9)	<.001^a^
Sex						
Male	87.0 (84.1-90.0)	[Reference]	81.0 (77.5-84.6)	[Reference]	69.7 (65.6-73.7)	[Reference]
Female	89.5 (86.8-92.1)	.23	81.6 (78.3-85.0)	.82	76.2 (72.5-79.9)	.02^a^
Age group, y						
18-29	89.2 (83.7-95.0)	[Reference]	78.5 (70.7-86.2)	[Reference]	66.6 (57.0-76.2)	[Reference]
30-44	86.9 (82.6-91.1)	.50	78.0 (73.2-82.7)	.91	70.1 (64.5-75.7)	.52
45-59	86.2 (82.0-90.4)	.40	83.0 (78.7-87.3)	.31	68.2 (62.7-73.8)	.77
≥60	90.2 (86.7-93.8)	.76	83.5 (78.6-88.3)	.31	80.0 (75.2-84.8)	.02^a^
Race and ethnicity						
Non-Hispanic or Hispanic Black	86.6 (83.0-90.2)	.39	82.4 (78.5-86.3)	.48	74.4 (69.8-79.0)	.49
Non-Hispanic White	88.5 (86.3-90.8)	[Reference]	80.7 (77.8-83.5)	[Reference]	72.3 (69.1-75.5)	[Reference]
Education						
<High school diploma	87.0 (78.7-95.3)	[Reference]	82.4 (72.9-92.0)	[Reference]	65.3 (50.2-80.5)	[Reference]
High school	88.9 (85.2-92.7)	.67	81.3 (76.6-86.1)	.83	67.9 (62.3-73.4)	.75
Some college	88.4 (84.3-92.4)	.77	82.7 (77.7-87.7)	.96	72.5 (66.9-78.2)	.39
≥Bachelor’s degree	87.8 (84.1-91.5)	.86	79.5 (75.3-83.7)	.60	77.3 (72.9-81.8)	.15
Household income, $						
<24 000	80.5 (72.8-88.2)	[Reference]	77.4 (69.9-85.0)	[Reference]	69.7 (60.7-78.7)	[Reference]
25 000-49 000	89.0 (84.3-93.7)	.05	79.1 (72.9-85.4)	.72	75.2 (68.8-81.7)	.30
50 000-74 999	86.6 (81.7-91.5)	.17	79.1 (73.4-84.9)	.72	77.3 (71.4-83.1)	.17
>75 000	90.5 (87.8-93.2)	.02	83.4 (79.8-86.9)	.19	70.8 (66.2-75.3)	.85
Employment status						
Unemployed	84.6 (76.6-92.6)	[Reference]	72.7 (62.3-83.0)	[Reference]	64.0 (51.5-76.5)	[Reference]
Employed	88.9 (86.2-91.5)	.32	80.7 (77.3-84.2)	.15	75.9 (72.3-79.6)	.08
Other	88.0 (84.0-92.1)	.48	83.5 (78.8-88.3)	.07	68.5 (62.4-74.5)	.54
Region						
Northeast	92.2 (88.4-95.9)	[Reference]	77.7 (71.5-83.8)	[Reference]	77.7 (71.7-83.7)	[Reference]
Midwest	86.9 (82.6-91.1)	.07	79.5 (74.3-84.6)	.66	71.6 (66.2-77.1)	.15
South	87.8 (84.6-90.9)	.08	82.8 (79.3-86.2)	.16	71.8 (67.3-76.3)	.12
West	87.2 (81.9-92.6)	.14	82.7 (76.7-88.7)	.25	71.1 (64.4-77.8)	.15
Experience with opioids						
No experience	85.0 (81.6-88.4)	[Reference]	79.3 (75.5-83.2)	[Reference]	67.9 (63.5-72.4)	[Reference]
Personal experience^b^	90.4 (88.0-92.8)	.01^a^	82.2 (79.1-85.3)	.26	75.9 (72.4-79.3)	.01^a^

^a^ Statistical significance at *P* < .05 level. The results in this table are average predicted probabilities estimated from multivariate logistic regression.

^b^ Personal experience includes self-reported experience with one’s own, a family member’s, or a close friend’s opioid addiction or overdose.

**Table 2.** Adjusted Attitudes About People With Opioid Addiction Among 1552 US Adults

Characteristic	Unwilling to have a person with opioid addiction as a neighbor		Unwilling to have a person with opioid addiction marry into the family	
	% (95% CI)	*P* value	% (95% CI)	*P* value
Political ideology				
Conservative	51.0 (45.7-56.3)	[Reference]	69.5 (64.6-74.4)	[Reference]
Moderate	34.9 (29.7-40.0)	<.001^a^	56.1 (50.8-61.3)	<.001^a^
Liberal	27.0 (21.9-32.0)	<.001^a^	47.4 (41.4-53.3)	<.001^a^
Sex				
Male	41.5 (37.3-45.7)	[Reference]	58.8 (54.5-63.0)	[Reference]
Female	35.1 (30.9-39.4)	.04^a^	57.2 (52.9-61.5)	.62
Age group, y				
18-29	49.3 (39.9-58.6)	[Reference]	64.6 (55.5-73.8)	[Reference]
30-44	41.0 (35.0-47.0)	.13	58.2 (52.5-63.9)	.23
45-59	34.1 (28.5-39.7)	.01^a^	54.7 (48.9-60.5)	.07
≥60	35.0 (29.3-40.7)	.02^a^	58.2 (52.0-64.3)	.28
Race and ethnicity				
Non-Hispanic or Hispanic Black	32.4 (27.3-37.5)	.03^a^	51.5 (46.2-56.9)	.02^a^
Non-Hispanic White	39.6 (36.1-43.0)	[Reference]	59.7 (56.2-63.2)	[Reference]
Education				
<High school diploma	30.2 (15.0-45.4)	[Reference]	45.0 (28.6-61.5)	[Reference]
High school	36.6 (31.0-42.2)	.43	58.5 (52.7-64.3)	.12
Some college	42.4 (36.5-48.2)	.14	60.6 (54.6-66.5)	.08
≥Bachelor’s degree	38.2 (33.1-43.3)	.34	58.1 (53.0-63.2)	.15
Household income, $				
<24 000	38.3 (28.8-47.8)	[Reference]	50.3 (40.7-59.9)	[Reference]
25 000-49 000	32.2 (25.2-39.3)	.30	55.5 (48.1-63.0)	.38
50 000-74 999	36.7 (30.0-43.4)	.79	55.4 (48.4-62.4)	.40
>75 000	41.0 (36.4-45.6)	.64	62.3 (57.8-66.9)	.04
Employment status				
Unemployed	40.1 (27.3-53.0)	[Reference]	53.3 (40.2-66.4)	[Reference]
Employed	34.7 (30.5-38.9)	.43	55.4 (50.9-59.8)	.77
Other	44.2 (38.2-50.3)	.58	64.2 (58.3-70.0)	.14
Region				
Northeast	41.6 (34.7-48.5)	[Reference]	59.4 (52.5-66.3)	[Reference]
Midwest	39.6 (33.5-45.7)	.67	58.6 (52.5-64.7)	.87
South	40.1 (35.3-44.8)	.72	58.8 (54.1-63.5)	.88
West	28.9 (22.1-35.7)	.01^a^	54.9 (47.1-62.6)	.39
Experience with opioids				
No experience	41.0 (36.3-45.7)	[Reference]	62.1 (57.5-66.8)	[Reference]
Personal experience^b^	36.5 (32.7-40.3)	.15	55.5 (51.5-59.4)	.03^a^

^a^ Statistical significance at *P* < .05 level. The results in this table are average predicted probabilities estimated from multivariate logistic regression.

^b^ Personal experience includes self-reported experience with one’s own, a family member’s, or a close friend’s opioid addiction or overdose.

Consistent with unadjusted findings, moderates and liberals were more likely than conservatives to view federal, state, and local governments as responsible for reducing opioid overdose deaths (eAppendix 3 in Supplement 1) and less likely to desire social distance from people with opioid addiction (Table 2). Adjusting for sociodemographic characteristics, an estimated 51.0% (95% CI, 45.7%-56.3%) and 69.5% (95% CI, 64.6%-74.4%) of conservatives were unwilling to have a person with opioid addiction as a neighbor or marry into their family, respectively, compared with 34.9% (95% CI, 29.7%-40.0%) and 56.1% (95% CI, 50.8%-61.3%) of moderates and 27.0% (95% CI, 21.9%-32.0%) and 47.4% (95% CI, 41.4%-53.3%) of liberals.

In multivariate analyses, personal experience of opioid addiction or overdose was associated with heightened perception of opioid overdose as a very serious problem among those with personal experience (90.4%; 95% CI, 88.0%-92.8%) vs those without (85.0% 95% CI, 81.6%-88.4%). There was also a greater attribution of responsibility for reducing overdose to pharmaceutical companies among those with personal experience (75.9%; 95% CI, 72.4%-79.3%) vs among those without (67.9%; 95% CI, 63.5%-72.4%). Female (76.2%; 95% CI, 72.5%-79.9%) relative to male (69.7%; 95% CI, 65.6%-73.7%) sex and being aged older than 60 years (80.0%; 95% CI, 75.2%-84.8%) relative to 18 to 29 years (66.6%; 95% CI, 57.0%-76.2%) were also associated with greater attribution of responsibility to pharmaceutical companies, and personal experience and Black race were associated with greater likelihood of attributing responsibility to federal, state, and local governments (eAppendix 3 in Supplement 1). Female (35.1%; 95% CI, 30.9%-39.4%) relative to male (41.5%; 95% CI, 37.3%-45.7%) sex; Black (32.4%; 95% CI, 27.3%-37.5%) relative to non-Hispanic White (39.6%; 95% CI, 36.1%-43.0%) race; and age groups 45 to 59 years (34.1%; 95% CI, 28.5%-39.7%) and 60 years or older (35.0%; 95% CI, 29.3%-40.7%) relative to 18 to 29 years (49.3%; 95% CI, 39.9%-58.6%) were associated with lower unwillingness to have a person with opioid addiction as a neighbor. Black race (51.5%; 95% CI, 46.2%-56.9%) relative to White race (59.7% 95% CI, 56.2%-63.2%) and personal experience with opioid addiction or overdose (55.5%; 95% CI, 51.5%-59.4%) relative to no experience (55.5%; 95% CI, 51.5%-59.4%) were associated with lower unwillingness to have a person with opioid addiction marry into the family.

## Discussion

In this survey study conducted in April 2025, 65% or more of conservatives, moderates, and liberals surveyed viewed opioid overdose as a serious problem and identified people who use opioids and pharmaceutical companies as responsible for solutions. Perceptions of problem severity were consistent with those from national surveys using similar (though not identical) items in the mid to late 2010s,^1^ suggesting that declines in opioid overdose deaths have not changed US residents’ views on this dimension.

More than 80% of respondents reported that people who use opioids are responsible for solving the overdose problem, consistent with the public’s views in 2014.^18^ A slightly higher proportion of respondents viewed pharmaceutical companies as responsible for reducing opioid overdose in the present 2025 survey (73%) than in 2014 (65%),^18^ a finding potentially due to public attention to the national opioid settlements. Where conservatives and moderates viewed people using opioids as most responsible for reducing overdose deaths, followed by pharmaceutical companies, liberals perceived pharmaceutical companies as most responsible. Greater attribution of responsibility to the group affected by the problem is associated with lesser support for policy solutions conferring benefits (eg, expanded treatment access) to those impacted.^19,20^

Overall, nearly 40% and 60% of respondents were unwilling to have a person with opioid addiction as a neighbor or marry into their family, respectively. While these findings indicate high public stigma, the percentage of respondents who reported unwillingness on the marry measure in 2025 was somewhat lower than the 68% of respondents reporting unwillingness to have a person with prescription opioid use disorder marry into their family in 2014 (a neighbor measure comparison is not available).^7^ This difference could be attributable to variation in study samples or item wording, or it may indicate impacts of ongoing efforts to destigmatize addiction as a treatable health condition. Prior-decade comparisons by political ideology are not available, but it is worth noting that in 2025, unwillingness on both measures was more than 20 percentage points lower among liberals than conservatives. Higher public stigma is positively correlated with preferences for punitive as opposed to public health–oriented policies to address opioid overdose.^7^

Political conservatives’, moderates’, and liberals’ perceptions of the severity of opioid overdose problem, responsibility for solving it, and people experiencing opioid addiction were similar in unadjusted and adjusted analyses, suggesting that differing views by ideology were not due to underlying differences in the sociodemographic characteristics measured. Beyond political ideology, few characteristics were associated with perceptions of the severity of the problem and responsibility for solving it. Personal experience with opioid addiction or overdose was correlated with heightened perception of severity and attribution of responsibility to pharmaceutical companies. This study’s findings that Black race and personal experience of opioid addiction or overdose were associated with less desire for social distance from a person with opioid addiction are consistent with findings from other recent studies.^11,21^

Prior research suggests that the views captured in this study may impact policy preferences.^6,7,9^ In a national survey of US adults conducted in 2020,^9^ greater stigmatizing attitudes toward people with opioid use disorder were correlated with lower support for expanding Medicaid for low-income families to cover addiction treatment, increasing government spending to improve treatment of opioid use disorder, and making the opioid overdose reversal agent naloxone available without a prescription. Relative to respondents identifying as Republican, Democrats had greater support for all of these policy options.^9^ We found that across ideological groups, non-Hispanic and Hispanic Black and non-Hispanic White US adults surveyed in this study endorsed people who use opioids and pharmaceutical companies as most responsible for reducing opioid overdose deaths. Fewer—between 45%-55%—endorsed federal, state, and local governments, which have led much of the response to date. Pharmaceutical companies are in the process of paying billions of dollars in opioid settlement funds to state and local governments, which will use the funding to support a variety of programs and services designed to curb addiction and overdose.^22^ These governments determine how the funds will be used and frequently partner with nonprofit organizations, such as after school programs or addiction clinics—which fewer than 30% of the adults surveyed viewed as responsible for reducing opioid overdose deaths—to implement these programs.

### Limitations

This study has limitations. The web survey may be subject to self-reporting and sampling biases. Although SSRS uses best-practice, probability-based recruitment to mitigate these issues, as noted previously, not all characteristics of the survey sample precisely aligned with national estimates. The social distance measures in this survey did not articulate whether the person with opioid addiction was actively experiencing symptoms of addiction, engaging in treatment, or in recovery; prior research has shown that US adults have heightened desire for social distance from people with symptomatic addiction and greater comfort with social closeness to people engaged in treatment or with a history of opioid use disorder.^21,23,24^ Prior work has shown differences in desire for social distance among people whose experience with opioid use disorder is their own personal experience vs a family member or close friend’s^21^; in the multivariate models, we collapsed these categories because when included separately, they dropped from the model due to collinearity. While we assessed a robust set of respondent sociodemographic characteristics, other unmeasured individual, interpersonal or area-level factors^21^ may correlate with political ideology and/or views about opioid overdose and addiction. Because this survey was embedded in a larger study designed to compare views among non-Hispanic and Hispanic Black and non-Hispanic White adults, results represent these groups only.

## Conclusions

In this study, US adults continued to view opioid overdose as a serious problem. Different views on the responsibility of individuals using opioids for addressing overdose and levels of public stigma may underlie varying preferences for future actions to curb overdose across political ideological groups.

## Notes

Supplement 1. eAppendix 1. Survey Instrument eAppendix 2. Weighted and Unweighted Demographic Characteristics of the Study Sample and National Rates in the American Community Survey and the American National Election Studies Survey eAppendix 3. Adjusted Perceptions of the Responsibility of Family Members, Government, and Nonprofits for Reducing Opioid Overdose Deaths

Supplement 2. Data Sharing Statement
