# Error in Methods

**DOI:** 10.1001/jamanetworkopen.2026.17028

**Published:** 2026-05-13

In the Original Investigation titled “Public Views About Opioid Overdose and People With Opioid Use Disorder,”^1^ published January 16, 2026, there was an error in the Methods. In the Measures section of the Methods, it stated that Likert scale categories were combined for categories 4 and 5 (binary measure value 1) and 1 and 3 (binary measure value 0); this should have been 1, 2, and 3 (ie, including Likert category 2). This article has been corrected.^1^
