# Supplementary material for: Public Views About Opioid Overdose and People With Opioid Use Disorder
Source: JAMA Netw Open. 2026 Jan 16;9(1):e2554314. doi: 10.1001/jamanetworkopen.2025.54314 (PMC12811807; doi:10.1001/jamanetworkopen.2025.54314)
Supplement: Supplement 1. — eAppendix 1. Survey Instrument eAppendix 2. Weighted and Unweighted Demographic Characteristics of the Study Sample and National Rates in the American Community Survey and the American National Election Studies Survey eAppendix 3. Adjusted Perceptions of the Responsibility of Family Members, Government, and Nonprofits for Reducing Opioid Overdose Deaths [file jamanetwopen-e2554314-s001.pdf]

Supplemental Online Content

McGinty EE, Gutkind S, Niederdeppe J, Franklin Fowler E, Barry CL. Public views about opioid overdose and people with opioid use disorder. *JAMA Netw. Open*. 2026;9(1):e2554314. doi:10.1001/jamanetworkopen.2025.54314

eAppendix 1. Survey Instrument

eAppendix 2. Weighted and Unweighted Demographic Characteristics of the Study Sample and National Rates in the American Community Survey and the American National Election Studies Survey

eAppendix 3. Adjusted Perceptions of the Responsibility of Family Members, Government, and Nonprofits for Reducing Opioid Overdose Deaths

This supplemental material has been provided by the authors to give readers additional information about their work.

© 2026 McGinty EE et al. *JAMA Network Open*.

eAppendix 1. Survey Instrument

This survey study is part of a larger message testing study focused on testing how different messages impacted views among non-Hispanic White adults relative to Black adults. This study analyzed data from the control arm of respondents who were not exposed to messaging and only answered the survey items.

The larger study included survey questions on two topics: opioids and Medicaid. Respondents were randomly assigned to answer either opioid items or Medicaid items first. The opioid section included a preamble introducing state laws requiring addiction treatment programs to offer medication for opioid use disorder. This preamble provided background information for survey items related to these laws that are not included in the present study.

Survey opioid preamble and items included in the present study

The FDA has approved three medications for treating opioid addiction. These medications are methadone, buprenorphine, and naltrexone. State laws requiring addiction treatment programs to offer FDA-approved medications for opioid addiction are designed to ensure that effective treatment is available to help people recover and to reduce opioid overdose deaths. A handful of states have passed these laws requiring addiction treatment programs to offer FDA-approved medications directly or coordinate with another program to provide medication access for their patients. Other states are now considering passing laws to require addiction treatment programs in their state to offer FDA-approved medications.

Please indicate your level of agreement with the following statement about opioid overdose deaths:

Primary measures

1. The number of people who die from opioid overdose in the U.S. is a very serious problem. (1=strongly disagree, 2=disagree, 3=neither agree nor disagree, 4=agree, 5=strongly agree)

2. How much responsibility do you believe each of the following groups should have for reducing opioid overdose deaths in the U.S.? (1=none at all, 2=a little bit, 3=some, 4=a lot, 5=a great deal)
    a. The federal government
    b. State governments
    c. Local governments
    d. Pharmaceutical companies
    e. Charitable organizations or nonprofits
    f. People who use opioids themselves
    g. Family members of people who use opioids

How willing would you be to…(1=definitely willing, 2=probably willing, 3=neither willing nor unwilling, 4=probably unwilling, 5=definitely unwilling)

3. Have a person with opioid addiction as a neighbor?
4. Have a person with opioid addiction marry into your family?

Socio-demographic characteristics

1. What is your age? Please type your age________________________

2. How do you describe your sex? Male/female/I describe myself another way/don't know/prefer not to answer

© 2026 McGinty EE et al. *JAMA Network Open*.

3. How do you describe your race?

    a. White/Black or African American/Asian, Native Hawaiian or Other Pacific Islander, Another Race/Refuse (note that only individuals identifying as White or Black or African American were eligible for this study)

4. Are you of Hispanic, Latino/a/x, or Spanish origin?
    a. Yes/no/refuse (note that that individuals who identified their race as White and their ethnicity as Hispanic/Latino were not eligible for this study)

5. What is the highest level of school you have completed or the highest degree you have received?
    a. Less than high school (Grades 1-8 or no formal schooling)/ high school incomplete (Grades 9-11 or Grade 12 with NO diploma)/ high school graduate (Grade 12 with diploma or GED certificate or vocational/ business technical or other training/ some college, no degree (includes some community college)/ two year associate degree from a college or university/ four year college or university degree (Bachelor's degree (e.g., BS, BA, AB)/ some postgraduate or professional schooling, no postgraduate degree/ postgraduate or professional degree, including master's, doctorate, medical or law degree (e.g., MA, MS, PhD, MD, JD)/ don't know; refuse/ prefer not to answer

6. What was the total combined income of all people in your household, including you, for the last 12-months?
    a. $0-$24,999/ $25,000-$49,999/ $50,000-$74,999/ $75,000-$99,999/ $100,000-$124,999/ $125,000-$149,999/ $150,000 or more/ refuse

7. Which of these best describes your employment status?
    a. Employed full-time (36 or more hours weekly) / unemployed/ retired/ student only/ student who is also employed/ full-time homemaker with no outside employment or student status/ employed part-time (fewer than 36 hours weekly) / other (please specify) / disabled or handicapped/ refuse

8. Generally speaking, do you think of yourself as a….
    a. Republican/Democrat/Independent/Another Party/No Preference/Refuse

9. In general, do you think of yourself as…
    a. Extremely liberal/liberal/slightly liberal/moderate, middle of the road/slightly conservative/conservative/extremely conservative

10. Have you every had an opioid addiction? No/yes/don't know/refuse

11. Have you ever had a family member or close friend who had an opioid addiction? No/yes/don't know/refuse

12. Have you personally known anyone who died from a drug overdose? No/yes/don't know/refuse

© 2026 McGinty EE et al. *JAMA Network Open*.

eAppendix 2. Weighted and Unweighted Demographic Characteristics of the Study Sample and National Rates in the American Community Survey and the American National Election Studies Survey

	National Comparison[1]	Unweighted	Weighted
Political Ideology			
Conservative	43.4	28.9	35.4
Moderate	21.8	39.6	35.3
Liberal	33.5	31.5	29.3
Female (%)	51.2	60.5	51.8
Age (%)			
18-29	18.4	11.3	14.1
30-44	24.4	33.7	27.7
45-59	22.7	27.4	25.0
60+	34.4	27.6	33.1
Mean age (range)	50 (18-99)	48.6 (19-95)	49.8 (19-95)
Race/Ethnicity (%)			
Non-Hispanic White	84.2	51.3	82.4
Non-Hispanic or Hispanic Black	15.8	48.7	17.6
Education (%)			
Less than High School Diploma	7.3	3.5	5.2
High School	27.5	25.1	28.8
Some college	30.2	30.3	26.4
Bachelor’s degree or higher	35.0	40.5	39.2
Household income (%)			
Under $24,999	13.7	13.6	12.2
$25,000- $49, 999	13.9	21.2	18.5
$50,000- $74,999	14.8	20.2	19.7
>$75,000	57.6	44.7	49.3
Employment status (%)			
Employed	60.9	63.1	58.4
Unemployed	2.5	6.8	6.9
Other	36.6	30.1	34.8
Region (%)			
Northeast	17.8	17.9	18.3
Midwest	23.6	21.3	25.0
South	40.1	47.1	40.4
West	18.5	13.7	16.3
Political Party Affiliation (%)			
Republican	45.5	28.8	42.2
Independent	8.0	17.3	13.3
Democrat	46.2	53.9	44.5
Experience with opioid addiction and overdose			
Personal experience with addiction	N/A	6.3	5.4
Family experience with addiction	N/A	43.7	44.8
Know someone who has died of an overdose	N/A	42.2	43.4

[1]Among non-Hispanic White and Black adults, aligning with our survey sample. Notes: National comparison data for demographic characteristics (i.e., sex, age, race and ethnicity, education, income, employment, and region) were obtained from the Current Population Survey (CPS) 2023 American Community Survey 1-Year Estimates using person-level weighted microdata (https://data.census.gov/app/mdat/ACSPUMS1Y2023). To be consistent with our survey sample, we estimated demographic characteristics among people who were 18 or older, and who identified as non-Hispanic White or Black. National comparison data for political party affiliation and political ideology were obtained from the American National Election Studies (ANES) 2024 Time Series Study Preliminary Release data (https://electionstudies.org/data-center/2024-time-series-study/), focusing on the fresh web sample that was designed to represent US citizens ages 18 or older living in 50 states and Washington, DC. In our sample, 8 people refused to report educational attainment (0.5% unweighted, 0.3% weighted), 4 people refused to report income (0.3% unweighted,

© 2026 McGinty EE et al. *JAMA Network Open*.

0.4% weighted). In the ANES sample 8 people in the fresh web sample refused the pre-election political party question (0.3%) and 21 people refused the pre-election political ideology questions (1.34%)

© 2026 McGinty EE et al. *JAMA Network Open*.

eAppendix 3: Adjusted Perceptions of the Responsibility of Family Members, Government, and Nonprofits for Reducing Opioid Overdose Deaths

Table 3.1: Adjusted perceptions of the responsibility of family members of people who use opioids for reducing opioid overdose among U.S. adults (N=1,552)

	**Family members of people who use opioids are responsible for reducing opioid overdose deaths**		
	% a lot or a great deal of responsibility	95% CI	p-value
Political ideology			
Conservative	56.5	51.0-62.0	Ref
Moderate	52.1	47.0-57.2	0.26
Liberal	46.0	40.3-51.7	0.01*
Sex			
Male (Ref)	50.1	45.7-54.6	Ref
Female	53.7	49.3-58.1	0.27
Age Group			
18-29 (Ref)	42.5	32.7-52.3	Ref
30-44	43.1	37.0-49.1	0.92
45-59	52.9	46.9-59.0	0.07
≥60	62.3	56.3-68.3	<0.001*
Race/Ethnicity			
Non-Hispanic White (Ref)	51.3	47.7-55.0	Ref
Non-Hispanic or Hispanic Black	54.3	49.0-59.7	0.38
Education			
Less than High School Diploma	53.0	38.5-67.5	Ref
High School	50.3	44.1-56.4	0.73
Some College	55.0	48.8-61.2	0.81
Bachelor's Degree or Higher	50.6	45.4-55.7	0.76
Household Income			
<$24,000	58.5	49.0-67.9	Ref
$25,000- $49,000	49.7	41.9-57.5	0.14
$50,000- $74,999	52.7	45.8-59.7	0.33
>$75,000	50.7	45.9-55.4	0.17
Employment Status			
Unemployed	57.6	45.2-69.9	Ref
Employed	52.4	48.0-56.7	0.44
Other	49.9	43.7-56.0	0.28
Region			
Northeast	50.4	43.3-57.5	Ref
Midwest	46.6	40.3-52.9	0.44
South	53.5	48.5-58.4	0.48
West	57.6	49.8-65.3	0.18
Experience with opioids			
No experience	51.5	46.6-56.4	Ref
Personal experience[1]	52.1	48.1-56.2	0.85

Note: Ref=reference group. Asterisk (*) indicates statistical significance at p<0.05 level. [1]Personal experience includes self-reported personal experience with opioid addiction, having a family member with opioid addiction, or knowing someone who has died of an opioid overdose.

© 2026 McGinty EE et al. *JAMA Network Open*.

Table 3.2: Adjusted perceptions of the responsibility of the federal government for reducing opioid overdose among U.S. adults (N=1,552)

	**The federal government is responsible for reducing opioid overdose deaths**		
	% a lot or a great deal of responsibility	95% CI	p-value
Political ideology			
Conservative	43.3	38.0-48.6	Ref
Moderate	56.7	51.5-61.9	0.001*
Liberal	68.1	62.7-73.5	<0.001*
Sex			
Male (Ref)	53.3	49.0-57.6	Ref
Female	56.6	52.3-60.9	0.29
Age Group			
18-29 (Ref)	55.8	46.5-65.0	Ref
30-44	52.7	46.8-58.7	0.58
45-59	51.9	46.0-57.7	0.48
≥60	59.7	53.7-65.7	0.50
Race/Ethnicity			
Non-Hispanic White (Ref)	52.5	48.9-56.0	Ref
Non-Hispanic or Hispanic Black	68.5	63.7-73.4	<0.001*
Education			
Less than High School Diploma	61.4	46.0-76.7	Ref
High School	56.4	50.5-62.3	0.54
Some College	54.4	48.3-60.5	0.41
Bachelor's Degree or Higher	54.4	49.3-59.5	0.42
Household Income			
<$24,000	51.2	40.8-61.5	Ref
$25,000- $49,000	58.2	50.8-65.6	0.25
$50,000- $74,999	57.9	51.1-64.7	0.28
>$75,000	54.3	49.7-58.9	0.60
Employment Status			
Unemployed	59.2	47.6-70.7	Ref
Employed	56.3	51.8-60.7	0.65
Other	52.7	46.7-58.8	0.34
Region			
Northeast	63.8	57.0-70.6	Ref
Midwest	54.4	48.2-60.5	0.04*
South	52.2	47.4-57.0	0.01*
West	54.4	46.8-62.0	0.07
Experience with opioids			
No experience	49.1	44.4-53.8	Ref
Personal experience[1]	59.6	55.6-63.5	0.001*

Note: Ref=reference group. Asterisk (*) indicates statistical significance at p<0.05 level. [1]Personal experience includes self-reported personal experience with opioid addiction, having a family member with opioid addiction, or knowing someone who has died of an opioid overdose.

© 2026 McGinty EE et al. *JAMA Network Open*.

Table 3.3: Adjusted perceptions of the responsibility of state governments for reducing opioid overdose among U.S. adults (N=1,552)

	**State governments are responsible for reducing opioid overdose deaths**		
	% a lot or a great deal of responsibility	95% CI	p-value
Political ideology			
Conservative	44.2	38.7-49.6	Ref
Moderate	58.0	52.8-63.2	<0.001
Liberal	66.6	61.1-72.1	<0.001
Sex			
Male (Ref)	53.6	49.2-58.0	Ref
Female	57.2	52.9-61.5	0.26
Age Group			
18-29 (Ref)	56.3	46.7-65.8	Ref
30-44	53.7	47.7-59.6	0.64
45-59	52.6	46.8-58.4	0.51
≥60	59.2	53.1-65.3	0.63
Race/Ethnicity			
Non-Hispanic White (Ref)	53.3	49.7-56.9	Ref
Non- Hispanic or Hispanic Black	66.4	61.4-71.4	<0.001
Education			
Less than High School Diploma	59.0	43.3-74.7	Ref
High School	53.8	47.7-59.8	0.53
Some College	56.0	49.9-62.1	0.72
Bachelor's Degree or Higher	56.5	51.4-61.7	0.78
Household Income			
<$24,000	53.2	42.6-63.8	Ref
$25,000- $49,000	54.2	46.7-61.6	0.88
$50,000- $74,999	62.8	55.9-69.6	0.14
>$75,000	53.9	49.2-58.6	0.92
Employment Status			
Unemployed	60.4	49.0-71.8	Ref
Employed	56.4	51.9-60.8	0.52
Other	53.3	47.3-59.4	0.29
Region			
Northeast	61.7	54.8-68.6	Ref
Midwest	56.1	49.9-62.4	0.24
South	52.8	47.9-57.7	0.04
West	54.8	47.1-62.4	0.19
Experience with opioids			
No experience	49.1	44.3-53.8	Ref
Personal experience[1]	60.2	56.2-64.2	0.001

Note: Ref=reference group. Asterisk (*) indicates statistical significance at p<0.05 level. [1]Personal experience includes self-reported personal experience with opioid addiction, having a family member with opioid addiction, or knowing someone who has died of an opioid overdose.

© 2026 McGinty EE et al. *JAMA Network Open*.

Table 3.4: Adjusted perceptions of the responsibility of the local governments for reducing opioid overdose among U.S. adults (N=1,552)

	**Local governments are responsible for reducing opioid overdose deaths**		
	% a lot or a great deal of responsibility	95% CI	p-value
Political ideology			
Conservative	37.6	32.4-42.8	Ref
Moderate	47.0	41.8-52.2	0.01
Liberal	54.3	48.6-60.1	<0.001
Sex			
Male (Ref)	41.1	36.8-45.5	Ref
Female	49.8	45.4-54.1	0.01
Age Group			
18-29 (Ref)	46.0	36.6-55.5	Ref
30-44	46.0	40.0-52.0	0.99
45-59	43.8	38.2-49.4	0.69
≥60	47.3	41.2-53.3	0.84
Race/Ethnicity			
Non-Hispanic White (Ref)	42.6	39.0-46.1	Ref
Non- Hispanic or Hispanic Black	61.3	56.0-66.6	<0.001
Education			
Less than High School Diploma	51.9	36.2-67.7	Ref
High School	43.9	38.0-49.8	0.34
Some College	47.7	41.6-53.8	0.62
Bachelor’s Degree or Higher	45.4	40.3-50.4	0.45
Household Income			
<$24,000	45.4	35.9-54.8	Ref
$25,000- $49,000	46.0	38.7-53.2	0.92
$50,000- $74,999	52.7	45.6-59.7	0.22
>$75,000	43.2	38.5-47.9	0.71
Employment Status			
Unemployed	50.4	38.5-62.3	Ref
Employed	45.9	41.6-50.3	0.49
Other	44.9	39.1-50.7	0.42
Region			
Northeast	51.1	44.0-58.2	Ref
Midwest	44.5	38.3-50.7	0.17
South	43.3	38.5-48.0	0.07
West	48.5	40.9-56.0	0.62
Experience with opioids			
No experience	38.0	33.4-42.6	Ref
Personal experience[1]	51.5	47.4-55.5	<0.001

Note: Ref=reference group. Asterisk (*) indicates statistical significance at p<0.05 level. [1]Personal experience includes self-reported personal experience with opioid addiction, having a family member with opioid addiction, or knowing someone who has died of an opioid overdose.

© 2026 McGinty EE et al. *JAMA Network Open*.

Table 3.5: Adjusted perceptions of the responsibility of nonprofit organizations for reducing opioid overdose among U.S. adults (N=1,552)

	**Nonprofit organizations are responsible for reducing opioid overdose deaths**		
	% a lot or a great deal of responsibility	95% CI	p-value
Political ideology			
Conservative	24.8	20.2-29.4	Ref
Moderate	28.8	24.1-33.5	0.24
Liberal	26.7	21.6-31.8	0.60
Sex			
Male (Ref)	24.0	20.2-27.9	Ref
Female	29.0	25.1-33.0	0.08
Age Group			
18-29 (Ref)	26.6	18.3-35.0	Ref
30-44	20.9	16.1-25.7	0.23
45-59	24.1	19.1-29.2	0.61
≥60	34.2	28.2-40.2	0.16
Race/Ethnicity			
Non-Hispanic White (Ref)	25.3	22.1-28.5	Ref
Non-Hispanic or Hispanic Black	33.6	28.3-38.9	0.01
Education			
Less than High School Diploma	28.9	16.0-41.9	Ref
High School	25.1	20.0-30.3	0.59
Some College	27.0	21.5-32.5	0.79
Bachelor's Degree or Higher	27.6	22.9-32.4	0.86
Household Income			
<$24,000	32.0	23.0-40.9	Ref
$25,000- $49,000	25.4	19.1-31.8	0.23
$50,000- $74,999	31.0	24.5-37.5	0.86
>$75,000	24.3	20.2-28.4	0.15
Employment Status			
Unemployed	31.4	19.0-43.8	Ref
Employed	27.7	23.6-31.8	0.57
Other	24.7	19.9-29.6	0.33
Region			
Northeast	29.1	22.6-35.7	Ref
Midwest	28.3	22.5-34.1	0.84
South	24.6	20.4-28.8	0.25
West	27.5	20.5-34.5	0.74
Experience with opioids			
No experience	25.6	21.4-29.7	Ref
Personal experience[1]	27.7	24.0-31.4	0.45

Note: Ref=reference group. Asterisk (*) indicates statistical significance at p<0.05 level. [1]Personal experience includes self-reported personal experience with opioid addiction, having a family member with opioid addiction, or knowing someone who has died of an opioid overdose.

© 2026 McGinty EE et al. *JAMA Network Open*.
