# Supplementary material for: Public Views About Opioid Overdose and People With Opioid Use Disorder
Source: JAMA Netw Open. 2026 Jan 16;9(1):e2554314. doi: 10.1001/jamanetworkopen.2025.54314 (PMC12811807; doi:10.1001/jamanetworkopen.2025.54314)
Supplement: Supplement 2. — Data Sharing Statement [file jamanetwopen-e2554314-s002.pdf]

# Data Sharing Statement

McGinty. Public Views About Opioid Overdose and People With Opioid Use Disorder. *JAMA Netw Open*. Published January 16, 2026. doi:10.1001/jamanetworkopen.2025.54314

**Data**
**Data available:** Yes
**Data types:** Deidentified participant data, Data dictionary
**How to access data:** Request to corresponding author Emma McGinty, emm4010@med.cornell.edu
**When available:** With publication

**Supporting Documents**
**Document types:** None

**Additional Information**
**Who can access the data:** Anyone requesting the data
**Types of analyses:** Any purpose
**Mechanisms of data availability:** No special mechanisms. The data will be provided upon request.
